# MiR-499-5p protects cardiomyocytes against ischaemic injury *via* anti-apoptosis by targeting PDCD4

**DOI:** 10.18632/oncotarget.9597

**Published:** 2016-05-25

**Authors:** Yingqing Li, Jianhua Lu, Xueming Bao, Xifu Wang, Junhua Wu, Xiongbin Li, Weiqiang Hong

**Affiliations:** ^1^ Department of Emergency, Guangzhou First People's Hospital, Guangzhou Medical University, Guangzhou, People's Republic of China

**Keywords:** miR-499-5p, acute myocardial infarction, cardiomyocytes apoptosis, ischaemic injury, programmed cell death 4, Pathology Section

## Abstract

Recent studies have reported that miRNAs might play critical roles in acute myocardial infarction (AMI). The objective of this study is to investigate the role of miR-499-5p in AMI and its potential molecular mechanisms. The expression level of MiR-499-5p was remarkably decreased in the infarcted myocardial tissues and in the cultured neonatal rat cardiomyocytes induced by hypoxia. Overexpression or knockdown of miR-499-5p decreased or increased the apoptotic rates of cultured cardiomyocytes in vitro. In addition, ectopic overexpression of miR-499-5p in the rat AMI models with agomir reduced the myocardial infarct size through decreasing the cardiomyocytes apoptosis in the infarcted area of the rat hearts. PDCD4 (programmed cell death 4) was verified as a direct target of miR-499-5p by luciferase report assay, and ectopic overexpression or inhibition of miR-499-5p could inhibit or increase the PDCD4 expression at both the mRNA and protein levels. Furthermore, we found that ectopic overexpression of PDCD4 without miR-499-5p binding sites reversed miR-499-5p-mediated cardiomyocytes apoptosis. Together, these findings revealed the role of miR-499-5p in protecting the cardiomyocytes against apoptosis induced by AMI via its direct target PDCD4, which providing evidence for the miR-499-5p/PDCD4 pathway as a potential therapeutic target for patients with AMI.

## INTRODUCTION

Acute myocardial infarction (AMI) is a common cardiovascular event and, although the principal ischaemic episode is often not fatal, the damage to myocardial tissues that is caused by AMI can lead to the development of heart failure, even cardiogenic shock, or death [1]. For these reasons, AMI is still a major global cause of morbidity and mortality, and approximately 3~4 million people each year are estimated to suffer from AMI. Despite with current optimal treatments, AMI is still the number one killer and is the world's leading cause of death [1–2]. Therefore, better understanding of the molecular mechanisms involved in AMI is essential for the development of novel therapeutic approaches.

MiRNAs are a class of small noncoding RNA molecules of 19-25 nucleotides in length, and act as negative regulators of gene expression by base paring with the 3′UTR of their target genes and resulting in either modulation of translation efficiency or degradation of the mRNA [3–4]. Generally, a miRNA can target multiple target genes, and one gene may be regulated by multiple miRNAs, underscoring the formation of complex regulatory networks [5–6]. Thus, it is not surprising that miRNAs can regulate diverse basic cellular functions including apoptosis and necrosis, which are two key cellular events in AMI [7–8]. Several recent researches have reported that the expression levels of some miRNAs were altered in the myocardium of rats with AMI [9–10]. It has also been demonstrated that these dysregulated miRNAs can regulate cardiomyocytes apoptosis and might be involved in the pathophysiology of AMI [11–19]. These results suggest that miRNAs play vital roles in myocardial apoptosis, however, the roles of miRNAs involved in myocardial apoptosis warrant further investigations.

As a cardiac-abundant miRNA under physiological conditions, miR-499-5p was found to be decreased in the infarcted area of rat heart by microarray analysis [9]. In addition, it was reported that the plasma level of miR-499-5p was significantly increased in AMI rats [20]. More interesting, this phenomena was verified in humans, and serum miR-499-5p could serve as a diagnostic biomarker for AMI patients based on our and other studies [20–24]. It has also been reported that miR-499-5p could regulate cardiomyocytes proliferation and differentiation [25–26], and it could protect cardiomyocytes from H_2_O_2_-induced apoptosis [27]. However, the potential effects of miR-499-5p treatment on myocardial infarct size in an *in vivo* AMI model and the mechanisms of its cardioprotection have not been investigated. Thus, the objective of this work is to study the role of miR-499-5p in AMI and its potential molecular mechanisms.

## RESULTS

### MiR-499-5p is reduced in infarcted myocardial tissues of AMI

**Figure 1:**
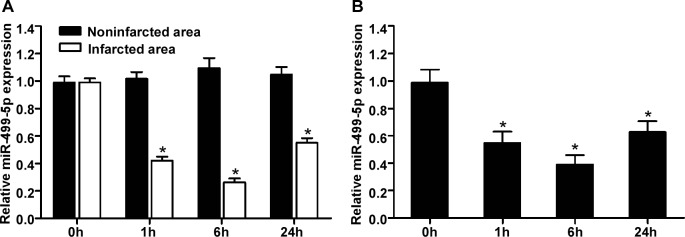
The expression levels of miR-499-5p in the infarcted hearts and neonatal rat cardiomyocytes under hypoxia **A.** Relative expression of miR-499-5p in the uninfarcted and infarcted areas of ischaemic heart was assessed by quantitative RT-PCR at the indicated times (n=6, each time-point per group). **B.** miR-499-5p expression in neonatal rat cardiomyocytes exposed to anoxia was analyzed at different time points. MiR-499-5p expression was normalized to U6 snRNA. Data was presented as the mean ± SD; *, *p* < 0.05.

To quantify the expression level of miR-499-5p in infarcted myocardial tissues of AMI rats, we isolated RNA from infarcted and noninfarcted myocardial tissues at 0 h, 1 h, 6 h, and 24 h after AMI. As shown in Figure 1A, compared with noninfarcted myocardial tissues, quantitative RT-PCR showed that the expression level of miR-499-5p was significantly downregulated in infarcted myocardial tissues at 1 h, 6 h, and 24 h after AMI (all *p* < 0.05), whereas its expression was not changed in the sham operated rats group (0 h).

### MiR-499-5p is reduced in cultured cardiomyocytes induced by hypoxia

To detect the effect of hypoxia on miR-499-5p expression in cultured cardiomyocytes, we collected RNA from cultured neonatal rat cardiomyocytes induced by hypoxia for 0 h, 1 h, 6 h, and 24 h. As shown in Figure 1B, quantitative RT-PCR demonstrated that the expression level of miR-499-5p was remarkably reduced in cardiomyocytes induced by hypoxia for 1 h, 6 h, and 24 h than those not induced by hypoxia (0 h) (all *p* < 0.05).

### MiR-499-5p inhibits cardiomyocytes apoptosis induced by hypoxia *in vitro*

**Figure 2:**
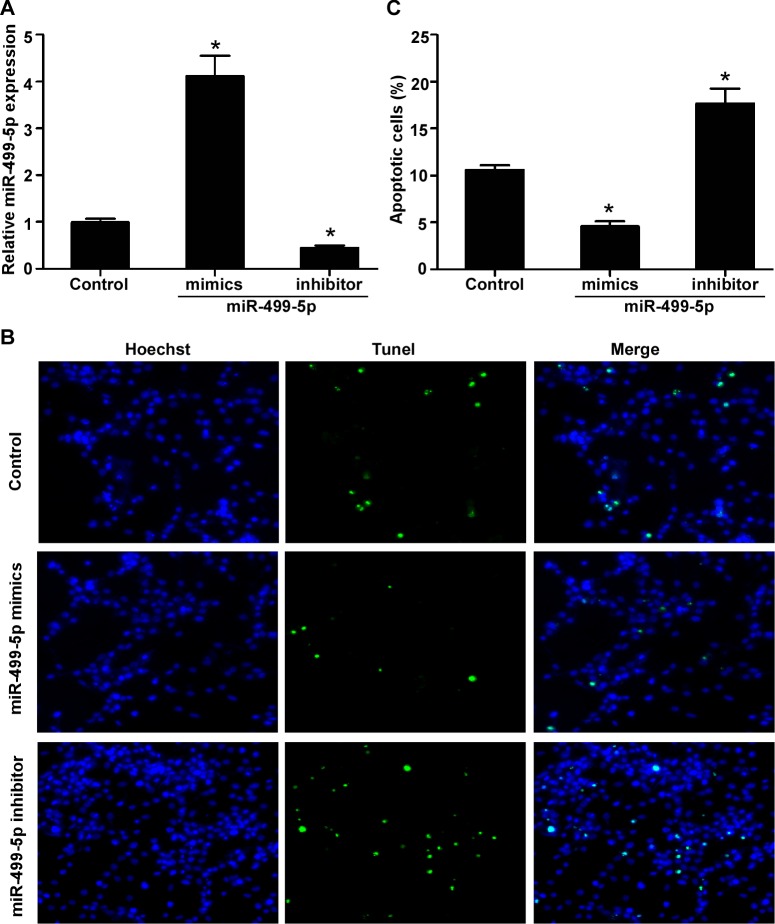
The effect of miR-499-5p on neonatal rat cardiomyocytes apoptosis under hypoxia **A.** Expression levels of miR-499-5p in cultured cardiomyocytes transfected with control, miR-499-5p mimics or inhibitor. **B.** Representative TUNEL-stained photomicrographs of cardiomyocytes treated with control, miR-499-5p mimics or inhibitor. **C.** Apoptotic rates of cardiomyocytes treated with control, miR-499-5p mimics or inhibitor. Data was presented as the mean ± SD; *, *p* < 0.05.

To demonstrate the effect of miR-499-5p on cardiomyocytes apoptosis, we transiently transfected the cultured neonatal rat cardiomyocytes with miR-499-5p mimics, inhibitor, or control, and then the cardiomyocytes were induced by hypoxia for 6 h. As shown in Figure 2A, transfection of miR-499-5p mimics or inhibitor could significantly increase or decrease the miR-499-5p expression level (*p* < 0.05). Representative TUNEL stained photomicrographs of cultured cardiomyocytes transfected with miR-499-5p mimics, inhibitor, or control were shown in Figure 2B. The results displayed that miR-499-5p mimics or inhibitor transfection could greatly inhibit or promote the cultured cardiomyocytes apoptosis induced by hypoxia compared with the control transfection (Figure 2C, *p* < 0.05).

### MiR-499-5p reduces the myocardial infarct size of AMI

**Figure 3:**
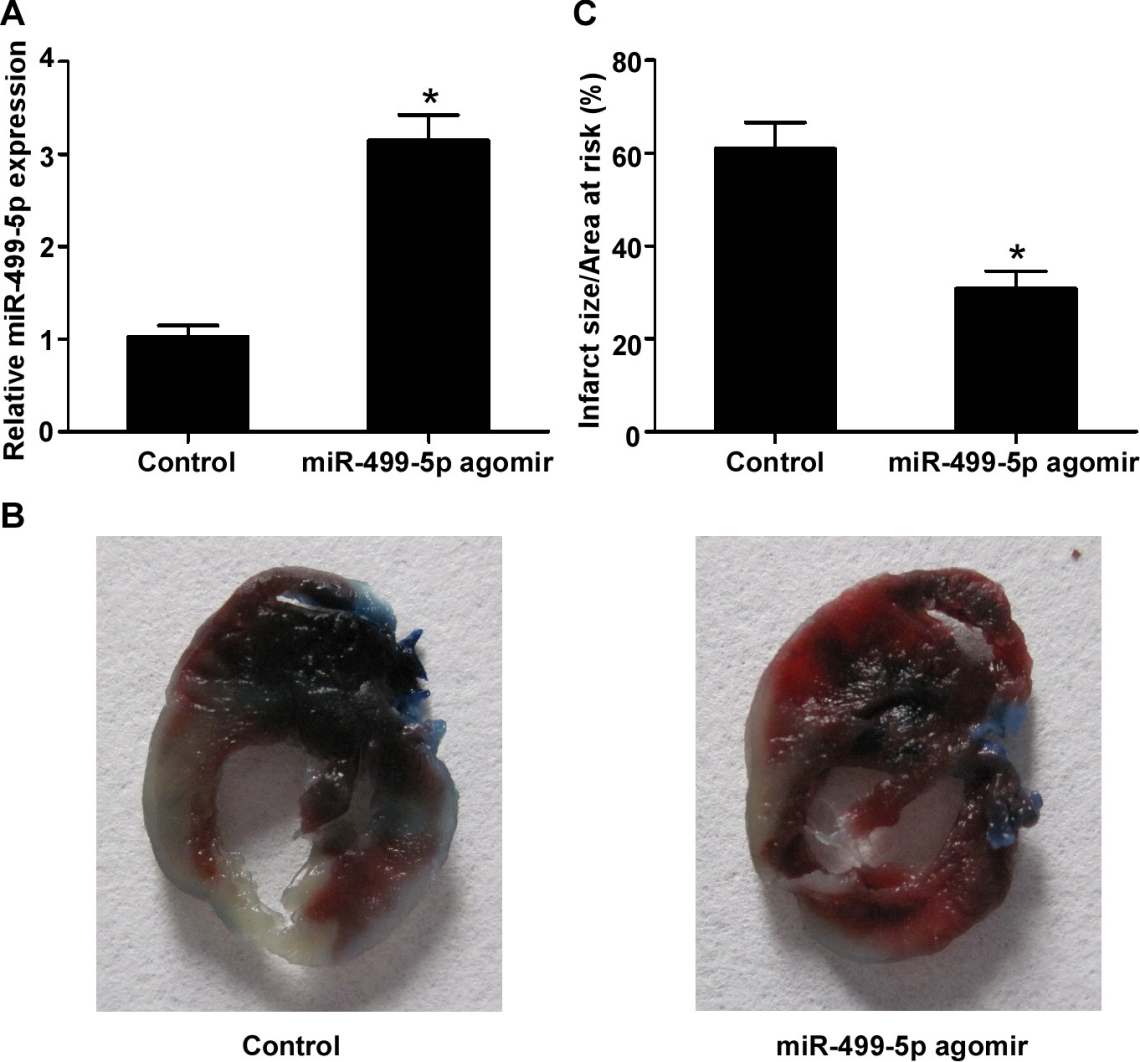
The effect of miR-499-5p overexpression on myocardial infarct size **A.** Expression levels of miR-499-5p in cardiomyocytes treated with control or miR-499-5p agomir. **B.** Representative TTC-stained heart slices from rats treated with control or miR-499-5p agomir. **C.** Infarcted size in rat hearts treated with control, miR-499-5p agomir or antagomir. Data was presented as the mean ± SD; *, *p* < 0.05.

To define the effect of miR-499-5p on the myocardial infarct size of AMI, we delivered the miR-499-5p agomir or control into the rat hearts to upregulate the miR-499-5p expression before the rat AMI models were established. As shown in Figure 3A, miR-499-5p agomir could remarkably enhance the expression level of miR-499-5p in the infarcted myocardial tissues of rats (*p* < 0.05). Representative TTC-stained heart slices from rats treated with miR-499-5p agomir or control were displayed in Figure 3B. The results showed that the myocardial infarct sizes were significantly inhibited by miR-499-5p agomir (Figure 3C, *p* < 0.05).

### MiR-499-5p inhibits cardiomyocytes apoptosis of AMI *in vivo*

**Figure 4:**
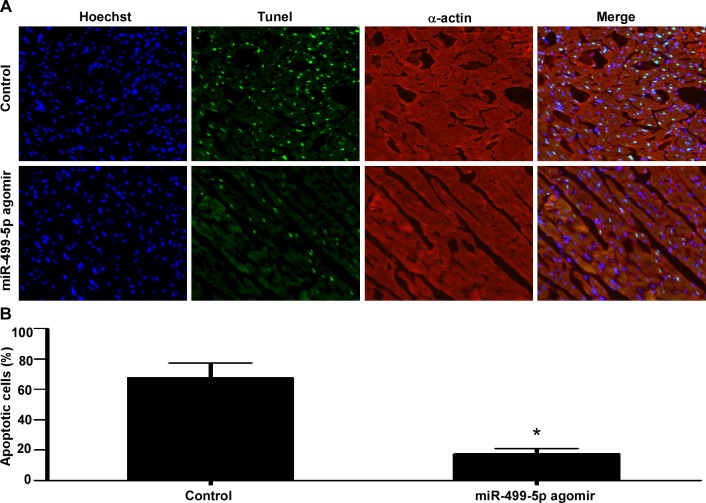
The effect of miR-499-5p on cardiomyocytes apoptosis in the infarcted hearts **A.** Representative TUNEL-stained photomicrographs of cardiomyocytes treated with control or miR-499-5p agomir. **B.** Apoptotic rates of cardiomyocytes treated with control or miR-499-5p agomir. Data was presented as the mean ± SD; *, *p* < 0.05.

To further define the potential cellular mechanism involved in miR-499-5p-mediated protective effects against myocardial infarction *in vivo*, we performed immunofluorescence with TUNEL staining to determine the apoptosis in infarcted rat heart sections treated with miR-499-5p agomir or control. Representative TUNEL stained photomicrographs of infarcted heart sections treated with miR-499-5p agomir or controls were displayed in Figure 4A. The results displayed that the apoptosis of cardiomyocytes in the infarcted area was significantly inhibited in the miR-499-5p agomir treated group than the control group (Figure 4B, *p* < 0.05).

### PDCD4 is verified as a direct target of miR-499-5p

**Figure 5:**
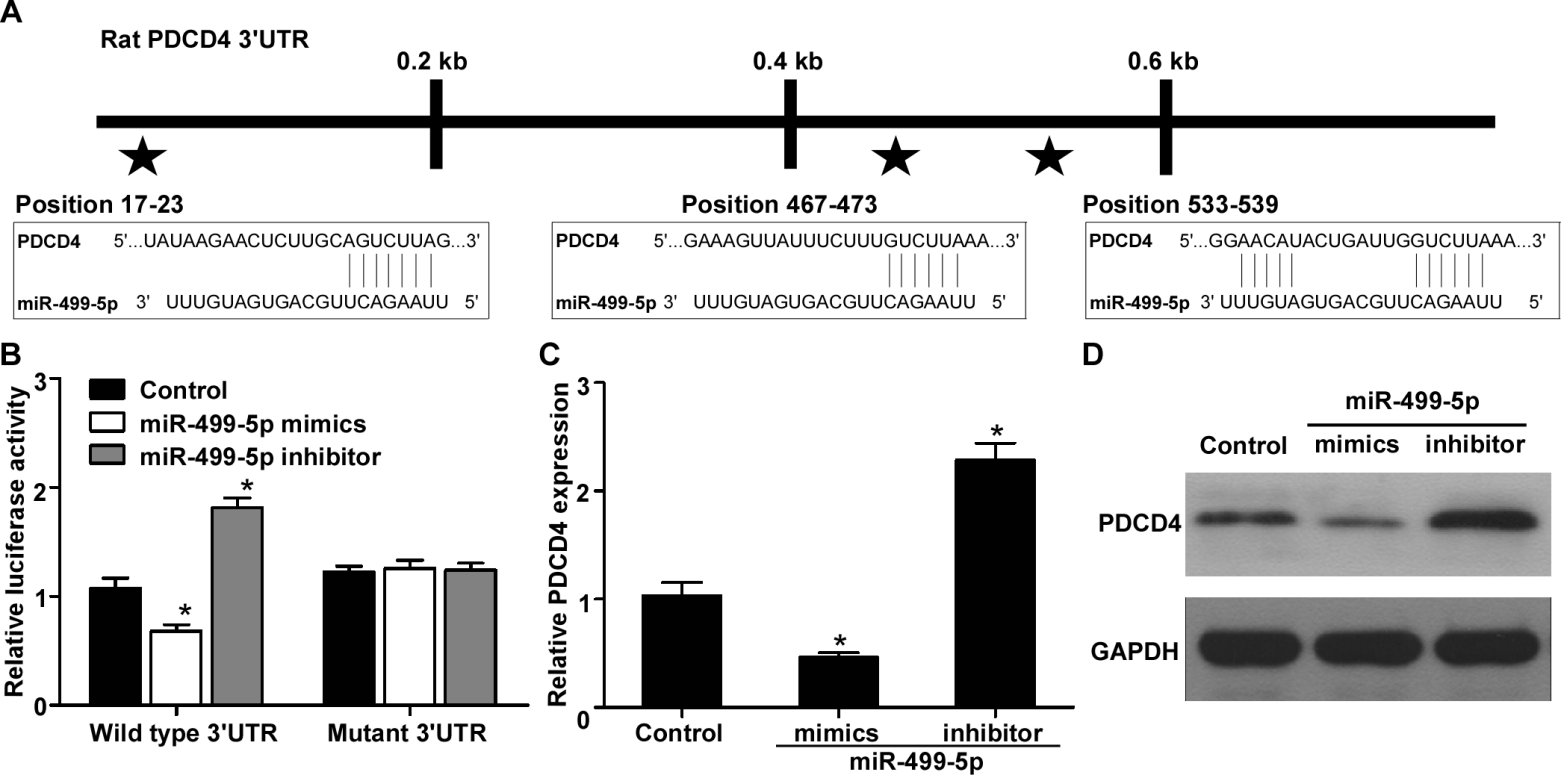
PDCD4 was verified as a direct target of miR-499-5p **A.** Three putative binding sites of miR-499-5p in the 3′UTR of PDCD4. **B.** Relative luciferase activity in cultured cardiomyocytes after cotransfected with wild type or mutant type 3′UTR and miR-499-5p mimics, inhibitor or control. **C.** Quantification of PDCD4 mRNA expression in cultured cardiomyocytes transfected with miR-499-5p mimics, inhibitor or control. **D.** Quantification of PDCD4 protein expression in cultured cardiomyocytes transfected with miR-499-5p mimics, inhibitor or control. Data was presented as the mean ± SD; *, *p* < 0.05.

To address the molecular mechanism of miR-499-5p involved in its protective effects against cardiomyocytes apoptosis, we searched for potential target genes of miR-499-5p with a publicly-available database TargetScan, and found that PDCD4 (programmed cell death 4) was predicted as a target of miR-499-5p as it has three potential binding sites within its 3′UTR (Figure 5A). The luciferase report assay confirmed that PDCD4 was a direct target of miR-499-5p because ectopic overexpression or inhibition of miR-499-5p could increase or decrease the luciferase activities of the wild type PDCD4 3′UTR reporter vector but not the mutant reporter vector (Figure 5B, P < 0.05). Furthermore, ectopic overexpression or inhibition of miR-499-5p could inhibit or increase the PDCD4 expression at both the mRNA and protein levels (Figure 5C-5D, *p* < 0.05).

### PDCD4 is involved in miR-499-5p-mediated cardiomyocytes apoptosis

**Figure 6:**
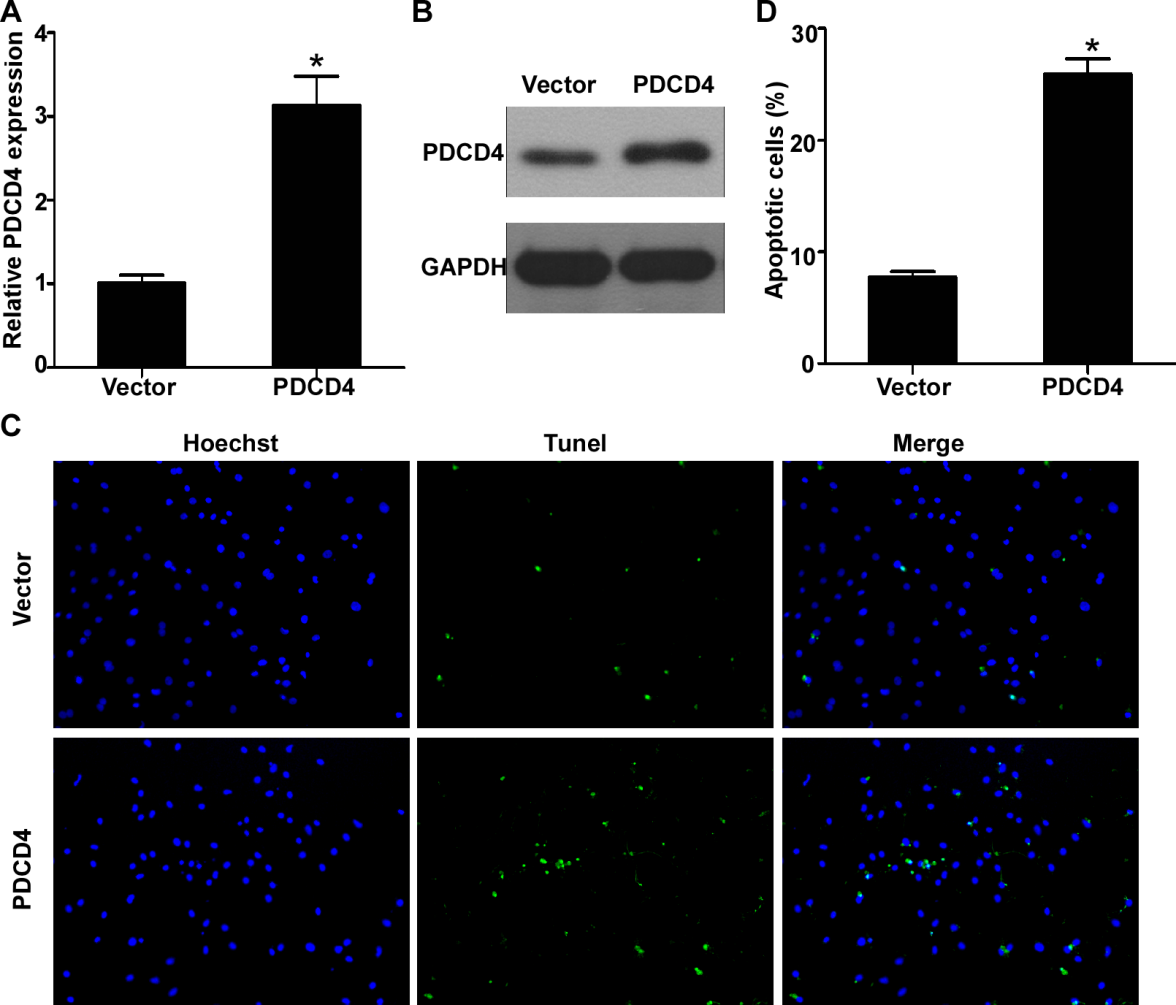
PDCD4 was involved in miR-499-5p-mediated cardiomyocytes apoptosis **A.** Expression levels of PDCD4 in cultured cardiomyocytes transfected with control or PDCD4 plasmid. **B.** Representative TUNEL-stained photomicrographs of cardiomyocytes treated with control or PDCD4 plasmid. **C.** Apoptotic rates of cardiomyocytes treated with control or plasmid. Data was presented as the mean ± SD; *, *p* < 0.05.

To demonstrate whether PDCD4 is involved in miR-499-5p-mediated cardiomyocytes apoptosis, we firstly transfected the cultured neonatal rat cardiomyocytes with miR-499-5p mimics to overexpress miR-499-5p, and then transfected it with PDCD4 plasmid or its empty vector. The PDCD4 plasmid encoded the full-length coding sequences of PDCD4 without its 3′UTR, thus could not be suppressed by miR-499-5p. Quantitative RT-PCR and western blotting showed that transfection of PDCD4 plasmid increased the mRNA and protein expression levels of PDCD4 (Figure 6A-6B, *p* < 0.05). Representative TUNEL stained photomicrographs of cultured cardiomyocytes transfected with PDCD4 plasmid or vector was displayed in Figure 6C. The results demonstrated that overexpression of PDCD4 could remarkably promote the cultured cardiomyocytes apoptosis induced by hypoxia (Figure 6D, *p* < 0.05).

## DISCUSSION

Up to now, AMI is still a major cause of morbidity and mortality worldwide. Apoptosis and necrosis are two key cellular events in AMI, and play important roles during the processes of AMI. Following the myocardial apoptosis, necrosis occurs during sustained and severe ischaemia, and it is an unregulated irreversible response to a fatal insult often involving the myocardial pump failure [28]. Therefore, reducing the magnitude of apoptosis and necrosis in myocardium may be the only way to prevent such mortality.

Recently, investigations about genes and gene products that involved in the regulation of myocardial apoptosis during AMI have been performed; however, the underlying molecular mechanism is still elusive. Increasing evidence has elucidated that in addition of genetic alteration, miRNAs also participate in the regulation of myocardial apoptosis [8]. It has been reported that the expression levels of some miRNAs were altered in the myocardium of AMI rats, and these dysregulated miRNAs can regulate the myocardial apoptosis [9–19]. Dong et al. reported that multiple miRNAs are aberrantly expressed in the early phase of AMI, and miR-21 has a protective effect on the myocardial infarction by reducing cardiac cell apoptosis *via* its target PDCD4 [9]. Ren et al. applied a well-established cardiac ischemia/reperfusion (I/R) model to determine the miRNA expression signature in ischemic hearts, and found that knockdown of endogenous miR-320 provided protection against I/R-induced cardiomyocyte death and apoptosis by targeting a well-studied cardioprotector HSP20 [10]. Xu et al. found that the muscle-specific miRNAs, miR-1 and miR-133, regulated myocardial apoptosis in an opposing action, with miR-1 being pro-apoptotic and miR-133 being anti-apoptotic [12]. In a word, miRNAs have a fundamental role in the pathophysiology of AMI.

In our present study, we focus on investigation of the role of miR-499-5p in AMI and its potential molecular mechanism. Based on previous microarray analysis, miR-499-5p was found to be downregulated in the infarcted area of rat heart [9]. We first established Rat AMI models and quantified the miR-499-5p expression in infarcted and noninfarcted myocardial tissues at 0 h, 1 h, 6 h, and 24 h after AMI using quantitative RT-PCR. Our results verified that the miR-499-5p level was obviously decreased in the infarcted myocardial tissues than noninfarcted myocardial tissues, suggesting that miR-499-5p in the non-infarcted area might participate in the pathophysiological response to AMI. We further detected the miR-499-5p expression in cultured neonatal rat cardiomyocytes induced by hypoxia, and found that the miR-499-5p level was remarkably reduced in cardiomyocytes induced by hypoxia than those not induced by hypoxia. Ectopic overexpression of miR-499-5p reduced the apoptotic rates of cardiomyocytes induced by hypoxia, whereas knockdown of endogenous miR-499-5p added the apoptotic rates of cardiomyocytes. In addition, overexpression of miR-499-5p in the rat models with agomir decreased the myocardial infarct sizes by reducing the cardiomyocytes apoptosis in the infarcted area of rat hearts. Taken together, these results demonstrated that miR-499-5p had a protective effect on myocardial infarction during AMI.

As we known, miRNA could exert their biological functions through modulating several target genes. The change of miR-499-5p expression level during the process of AMI might be important in the modulations of the expression of multiple genes and signaling transduction pathways. Recent studies have identified and verified several target genes of miR-499-5p, such as Sox6, Cyclin D1, calcineurin, dynamin-related protein-1, and phosphofurin acidic cluster sorting protein 2 [25, 27, 29]. In the current study, we predicted the potential target genes of miR-499-5p using computational bioinformatic analysis, and found that PDCD4 might be a miR-499-5p target. PDCD4 is a pro-apoptotic factor and plays important roles in regulating myocardial apoptosis [30–31]. PDCD4 has been validated as a direct target of miR-21, and it has been reported that miR-21 could protect myocardium against ischemia-induced or H_2_O_2_-induced apoptosis and ischaemia/reperfusion injury through targeting PDCD4 [9, 32–33]. In this study, the luciferase report assay also validated that PDCD4 was a direct target of miR-499-5p because ectopic overexpression or inhibition of miR-499-5p increased or decreased the luciferase activities of cardiomyocytes transfected with the wild type PDCD4 3′UTR reporter vector rather than the mutant reporter vector, which is also validated by two recent researches [34–35]. Furthermore, the mRNA and protein expression of PDCD4 in cardiomyocytes is able to be modulated by miR-499-5p as detected by both gain-of-function and loss-of-function strategies. In addition, we found that the protective effect of miR-499-5p overexpression on cardiomyocyte apoptosis induced by hypoxia was blocked after ectopic overexpression of PDCD4 without the miR-499-5p binding site. These results suggested that miR-499-5p could protect against myocardial apoptosis *via* its functional target PDCD4.

Our and several other recent studies have reported that miR-499-5p might leak out of the necrotic myocardium and into the circulation during early stages of AMI; furthermore, the level of circulating miR-499-5p decreases to normal level at the time of hospital discharge. Thus, the circulating miR-499-5p is useful as biomarker for the diagnosis of AMI [20–24]. Increasing evidence also demonstrated that the utilizing of miRNA expression alterations to influence the signal pathways had the potential of being translated into clinical applications [36–37]. Based on our present study, we would like to point out that there was still no enough data on modulating miR-499-5p to protect against cardiomyocytes apoptosis. Nevertheless, novel therapeutic strategies for AMI patients based on the miR-499-5p/PDCD4 pathway will be further investigated in our future study. In addition, each miRNA often has hundreds of target genes, thus, it is essential to explore the target network of miR-499-5p that involved in maintaining the cardiac function after AMI. This may lead to rationally select the targets for therapeutic intervention in patients suffering from AMI. Despite limitations, the current study represents for the first general research investigating the roles of miR-499-5p and PDCD4 in the pathophysiology of AMI and its potential molecular mechanisms.

In conclusion, our current work demonstrated that miR-499-5p was reduced in infarcted myocardial tissues of AMI and in cultured cardiomyocytes induced by hypoxia. Functional studies revealed that miR-499-5p could protect cardiomyocytes against apoptosis by directly targeting the pro-apoptotic factor PDCD4. Therefore, miR-499-5p has important role in the pathophysiology of AMI. The findings may help to further clarify the molecular mechanisms involved in the process of AMI, and provide evidence for the miR-499-5p/PDCD4 pathway as a potential therapeutic target for patients with AMI.

## MATERIALS AND METHODS

### Rat AMI model

Male Sprague-Dawley rats (200-250g) were provided by Medical Experimental Animal Center of Guangdong Province (Guangzhou, China). All procedures were in accordance with the rules of the Animal Care and Use Ethnic Committee of Guangzhou Medical University. Rat AMI model was established with left coronary artery ligation as described by Eckle et al [38]. Briefly, the rats AMI model was generated by occlusion of the left anterior descending coronary artery with a silk suture. Sham operated rats, which received an identical operation without the left coronary artery ligation, were used as controls.

### Identification of infarcted and noninfarcted areas

To define infarcted and noninfarcted areas, we firstly injected 1% Evans blue dye (3 ml) into the vena cava of rat, and then, the ventricles were cut transversely into 2-mm thick slices which incubated in 1% triphenyltetrazolium chloride (TTC). In myocardial slices, the region stained by the Evans blue was the noninfarcted area, the region unstained by the Evans blue and stained by the TTC was the border area, and the region unstained by both the Evans blue and TTC was the infarcted area. The infarcted size was measured by the Osiris software and calculated as follows: infarcted area / (infarcted area + border area).

### Neonatal rat cardiomyocytes culture

Neonatal rat cardiomyocytes were obtained and cultured as described by Dong et al [9]. In brief, the ventricles from Sprague-Dawley rats (1-2 days old) were minced with scissors, and then the ventricular cells were dispersed by digestion and cultured in cardiac myocyte culture medium. Hypoxia was achieved by culturing cardiomyocytes in a hypoxia chamber filled with 5% CO_2_ and 95% N_2_.

### Oligonucleotide and plasmids transfection

The miR-499-5p mimics, inhibitor, and control were purchased from GenePharma. The p-EZ-M02- PDCD4 plasmid and vector were purchased from FulenGen. After seeded into 6-well plates, cells were transfected with miR-499-5p mimics (50 nM), miR-499-5p inhibitor (100 nM), or PDCD4 plasmid (2 μg) using Lipofactamine 2000 reagent (Invitrogen) according to the manufacturer's instruction.

### RNA extraction and quantitative RT-PCR

Total RNA was extracted using TRIzol reagent (Invitrogen). The cDNA templates were obtained by M-MLV reverse transcriptase (Promega) and Bulge-Loop™ specific RT-Primers (RiboBio) for miR-499-5p or random primers (Promega) for PDCD4. Quantitative PCR reactions were done on the PRISM 7900HT sequence detection system (Applied Biosystems) using SYBR Green reagents (Invitrogen). The U6 or GAPDH were used for normalization, and the relative expression levels were calculated using 2^−ΔΔCt^ method.

### TUNEL assay

Apoptosis in cultured cardiomyocytes and heart sections were determined by TUNEL staining based on labeling of NDA strand breaks using the *In situ* Cell Death Detection Kit (Roche) according to the manufacturer's instruction. In brief, cardiomyocytes cultured on the coverslips in 24-well plates and frozen heart sections with 8μm thick were fixed with 4% paraformaldehyde for 1 h at 25°C, incubated in 0.1% Triton X-100 for 2 min on ice, and then incubated with 50 μl TUNEL reaction mixture for 1 h at 37°C in a chamber with humidified atmosphere and in dark. The numbers of TUNEL-positive cells and the total cells were counted under a fluorescence microscope. α

### Overexpression of miR-499-5p *in vivo*

MiR-499-5p agomir (RiboBio) was delivered into hearts to upregulate the miR-499-5p expression through a local delivery method as previously described [9]. Briefly, the aorta and pulmonary arteries were identified and clamped after anaesthetized and thoracotomy, and the solution containing miR-499-5p agomir (10 nM, RiboBio) or control dissolved in 200 μl PBS was injected through a catheter which injected from the apex of the left ventricle to the aortic root. After injection, the clamps were maintained for 10 s to make the solution circulate down the coronary arteries and perfuse the heart. Then, the clamps were released, the chests were closed, and the left coronary artery ligation was conducted after 24 h in these rats.

### Luciferase reporter assay

A fragment of the PDCD4 wild type 3′UTR containing the putative miR-499-5p binding sequence was cloned into a firefly luciferase reporter vector psiCHECK™ (Promega), and the PDCD4 mutant 3′UTR plasmid was subsequently generated by site-directed mutagenesis and used as a control. Then, the cardiomyocytes were co-transfected with the wild type or mutant PDCD4 3′UTR plasmid and miR-499-5p mimics, inhibitor, or control using Lipofectamine 2000 (Invitrogen), together with the Renilla luciferase-expressing vector pRL-TK (Promega) used as a spiked-in control to normalize the transfection efficiency. After transfection for 48 h, cells were harvested, and the luciferase activity was measured by a Dual Glo^TM^ Luciferase Assay System (Promega) according to the manufacturer's instructions.

### Western blot analysis

The total proteins was achieved from cultured cardiomyocytes using RIPA buffer with protease inhibitor Cocktail (Pierce), and the protein concentrations were determined using the BCA protein Assay Kit (Pierce). Equal amounts of protein were separated on the SDS-PAGE gels, and then transferred to PVDF membranes (Millipore) to analyze the expression levels of PDCD4 by incubating with rabbit polyclonal anti-PDCD4 antibody (1:1000, Abcam) followed by HRP-conjugated goat anti-rabbit antibody. An anti-GAPDH antibody (1:1000, Abcam) was used as a loading control to normalize the protein levels. The immunolabelling was detected using an enhanced chemiluminescence detection system.

### Statistical analysis

The Statistical Package for Social Sciences, version 16.0 (SPSS, Inc.) was used for all of the statistical analysis. All of the data was presented as mean ± SD, and differences between groups were analyzed using the Student's t-test. A two tailed p value of < 0.05 was defined as statistically significant.
